# TRPV1 and TRPV4 Play Pivotal Roles in Delayed Onset Muscle Soreness

**DOI:** 10.1371/journal.pone.0065751

**Published:** 2013-06-17

**Authors:** Hiroki Ota, Kimiaki Katanosaka, Shiori Murase, Makiko Kashio, Makoto Tominaga, Kazue Mizumura

**Affiliations:** 1 Department of Neural Regulation, Graduate School of Medicine, Nagoya University, Nagoya, Japan; 2 Department of Physical Therapy, College of Life and Health Sciences, Chubu University, Kasugai, Japan; 3 Department of Neuroscience II, Research Institute of Environmental Medicine, Nagoya University, Nagoya, Japan; 4 Division of Cell Signaling, Okazaki Institute for Integrative Bioscience, National Institute for Physiological Sciences, Okazaki, Japan; Hokkaido University, Japan

## Abstract

Unaccustomed strenuous exercise that includes lengthening contraction (LC) often causes tenderness and movement related pain after some delay (delayed-onset muscle soreness, DOMS). We previously demonstrated that nerve growth factor (NGF) and glial cell line-derived neurotrophic factor (GDNF) are up-regulated in exercised muscle through up-regulation of cyclooxygenase (COX)-2, and they sensitized nociceptors resulting in mechanical hyperalgesia. There is also a study showing that transient receptor potential (TRP) ion channels are involved in DOMS. Here we examined whether and how TRPV1 and/or TRPV4 are involved in DOMS. We firstly evaluated a method to measure the mechanical withdrawal threshold of the deep tissues in wild-type (WT) mice with a modified Randall-Selitto apparatus. WT, TRPV1−/− and TRPV4−/− mice were then subjected to LC. Another group of mice received injection of murine NGF-2.5S or GDNF to the lateral gastrocnemius (LGC) muscle. Before and after these treatments the mechanical withdrawal threshold of LGC was evaluated. The change in expression of NGF, GDNF and COX-2 mRNA in the muscle was examined using real-time RT-PCR. In WT mice, mechanical hyperalgesia was observed 6–24 h after LC and 1–24 h after NGF and GDNF injection. LC induced mechanical hyperalgesia neither in TRPV1−/− nor in TRPV4−/− mice. NGF injection induced mechanical hyperalgesia in WT and TRPV4−/− mice but not in TRPV1−/− mice. GDNF injection induced mechanical hyperalgesia in WT but neither in TRPV1−/− nor in TRPV4−/− mice. Expression of NGF and COX-2 mRNA was significantly increased 3 h after LC in all genotypes. However, GDNF mRNA did not increase in TRPV4−/− mice. These results suggest that TRPV1 contributes to DOMS downstream (possibly at nociceptors) of NGF and GDNF, while TRPV4 is located downstream of GDNF and possibly also in the process of GDNF up-regulation.

## Introduction

Delayed-onset muscle soreness (DOMS) is described as unpleasant sensation or tenderness after unaccustomed strenuous exercise [1]. In humans, it usually appears after some delay (up to 1–2 days), and disappears naturally within 3–7 days [1], [2]. The delay of appearance after exercise is mysterious and the reason was not known.

Recently, a model using lengthening contraction (LC) and a method for evaluating muscular mechanical hyperalgesia (tenderness) in rats have been developed [3]. Using these tools, two pathways for generating DOMS have been demonstrated: One is from bradykinin-like substance (arg-bradykinin in rats) to nerve growth factor (NGF) [4] and the other is from COX-2 to glial cell line-derived neurotrophic factor (GDNF) [5].

While NGF is the prototypical neurotrophic and survival factor for both sympathetic and sensory neurons during development [6], it is involved in inflammatory hyperalgesia in adulthood [7], [8]. Previous study from our laboratory has shown NGF is produced in the muscle after LC and plays a pivotal role in DOMS [4]. NGF is also produced in the skeletal muscle after ischemia [9] and nerve injury [10], and sensitizes muscular nociceptors [4], [11]. Intramuscular injection of NGF induces lasting tenderness in humans [12]. Similar to NGF, GDNF also works as a trophic factor for a subgroup of developing sensory neurons in fetal life [13] and is involved in several pain states in adult animals, but its effects are either analgesic [14], [15] or hyperalgesic [16].

Transient receptor potential (TRP) channels are sensitive to heat/warm (TRPV1 [17], TRPV2 [18], TRPV3 [19], TRPV4 [20]), cold (TRPM8[21]), acid (TRPV1 [22]), and various chemical substances (TRPV1 [22], TRPV2 [23], TRPV3 [24], TRPV4 [25], TRPM8 [21], and TRPA1 [26]), and are expressed not only in sensory neurons but also in keratinocytes (TRPV3 [24], TRPV4 [27]) and muscle fibers (TRPV4) [28]–[30]. Recently it is suggested that TRPs are involved in osmosensitivity and mechanosensitivity (see [31] for review). TRPV1 itself is not sensitive to mechanical stimulation, but its role not only in heat but also mechanical hyperalgesia induced by muscle inflammation has been reported [32]. Recent study from our group also indicated involvement of TRP channels in DOMS [33]. TRPV4 has been implicated in a wide variety of mechanosensory processes [34]–[36] and osmosensitivity [37], [38]. TRPV4 is expressed in muscle fibers; therefore, if it is involved in DOMS, it may be involved not only in sensitizing nociceptors but also in the mechanical event during LC leading to up-regulation of neurotrophins.

The purpose of this study is to investigate whether TRPV1 and/or TRPV4 is/are involved in DOMS and whether they work downstream or upstream of NGF or GDNF up-regulation, using TRPV1 and TRPV4 knockout mice. We found TRPV1 was involved only downstream of both NGF and GDNF up-regulation, while TRPV4 was involved downstream and possibly also in the process of GDNF up-regulation.

## Results

### Modified Randall-Selitto Method can Measure the Mechanical Withdrawal Threshold of Deep Tissues in Mice

**Figure 1:**
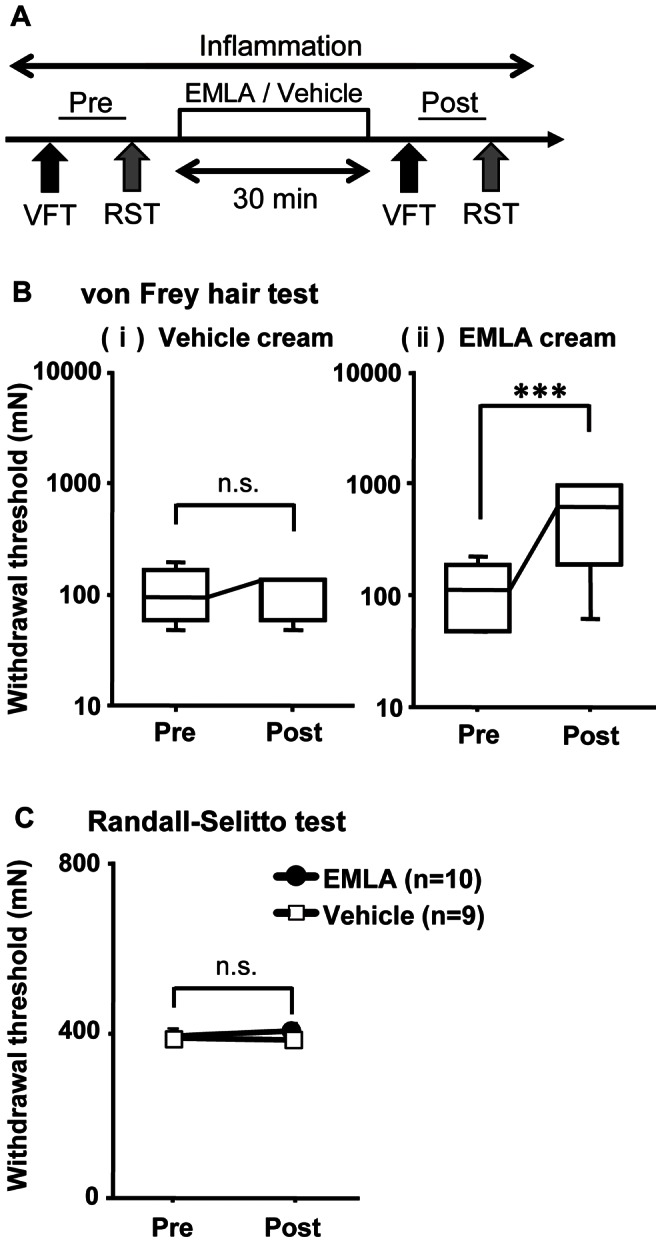
Measurement of muscular mechanical withdrawal threshold with Randall-Selitto apparatus in mice. (**A**) Schedule for testing effects of EMLA cream treatment on the withdrawal threshold. More than 12 h before these measurements, inflammation was induced by injecting carrageenan into the LGC muscle. VFT: von Frey hair test, RST: Randall-Sellito test. (**B**) Change in VFT threshold (tip diameter: 0.25 mm) by surface anesthesia. (i) Vehicle cream (n = 9) did not change the threshold. (ii) EMLA treatment (n = 10) significantly raised the threshold compared with before the treatment. Median and interquartile range (IQR) are shown. *** *p*<0.001 for pre- and post-cream treatment comparison by Mann-Whitney test. Note that pre values are decreased ones after induction of inflammation (same in C). (**C**) Change after surface anesthesia by EMLA cream treatment in withdrawal threshold measured by RST with a self-made larger probe (tip diameter: 2.6 mm). Filled circles: EMLA treatment (n = 10), open square: vehicle cream treatment (n = 9). EMLA cream did not significantly change the threshold, the same as vehicle cream. Mean ± S.E.M. (n = 6–10 for each group). S.E.M.s are hardly seen because they are small.

We examined whether the mechanical withdrawal threshold measured with a Randall-Selitto apparatus equipped with a larger probe (tip diameter: 2.6 mm) than commercially available one reflects deep (possibly muscle) mechanical withdrawal threshold in mice as it does in rats, and also in the presence of cutaneous punctate hyperalgesia. Similar to our previous experiment in rats [39], we induced cutaneous and muscular mechanical hyperalgesia by injecting carrageenan into the lateral gastrocnemius (LGC) muscle of the right side. The time schedule of carrageenan injection, measurement of withdrawal threshold, and local anesthetic treatment is shown in Fig. 1A. We confirmed that more than 12 h after the carrageenan injection, the von Frey hair test (VFT) threshold of the skin over the LGC muscle decreased. The median before carrageenan injection was 236.3 mN (interquartile range (IQR): 166.6–236.3 mN); that after the injection was 95.1 mN (IQR: 58.9–196.0 mN); *p*<0.001, Mann-Whitney test (n = 19). The Randall-Selitto test (RST) threshold was also significantly decreased (average before carrageenan injection 528.6±21.3 mN; after injection 381.6±13.4 mN, n = 19). The decreased VFT threshold was significantly reversed by application of a local anesthetic, EMLA®, (*p*<0.001, Mann-Whitney test; Fig. 1B (ii), n = 10), but that in the vehicle group was not (Fig. 1B(i), n = 9). In contrast, EMLA treatment induced no changes in the decreased deep mechanical threshold (EMLA group in Fig. 1C), the same as in the vehicle group (Fig. 1C). These results suggest that VFT with tip diameter of 0.25 mm measure the mechanical withdrawal threshold of skin, while the RST with a probe of 2.6 mm in diameter measures that of deep tissues, possibly muscle, even when the skin is hyperalgesic. Therefore, we used the Randall-Selitto apparatus equipped with this large probe in the following experiments to measure the muscle mechanical withdrawal threshold in mice.

### LC Induced DOMS in Wild-type Mice

**Figure 2:**
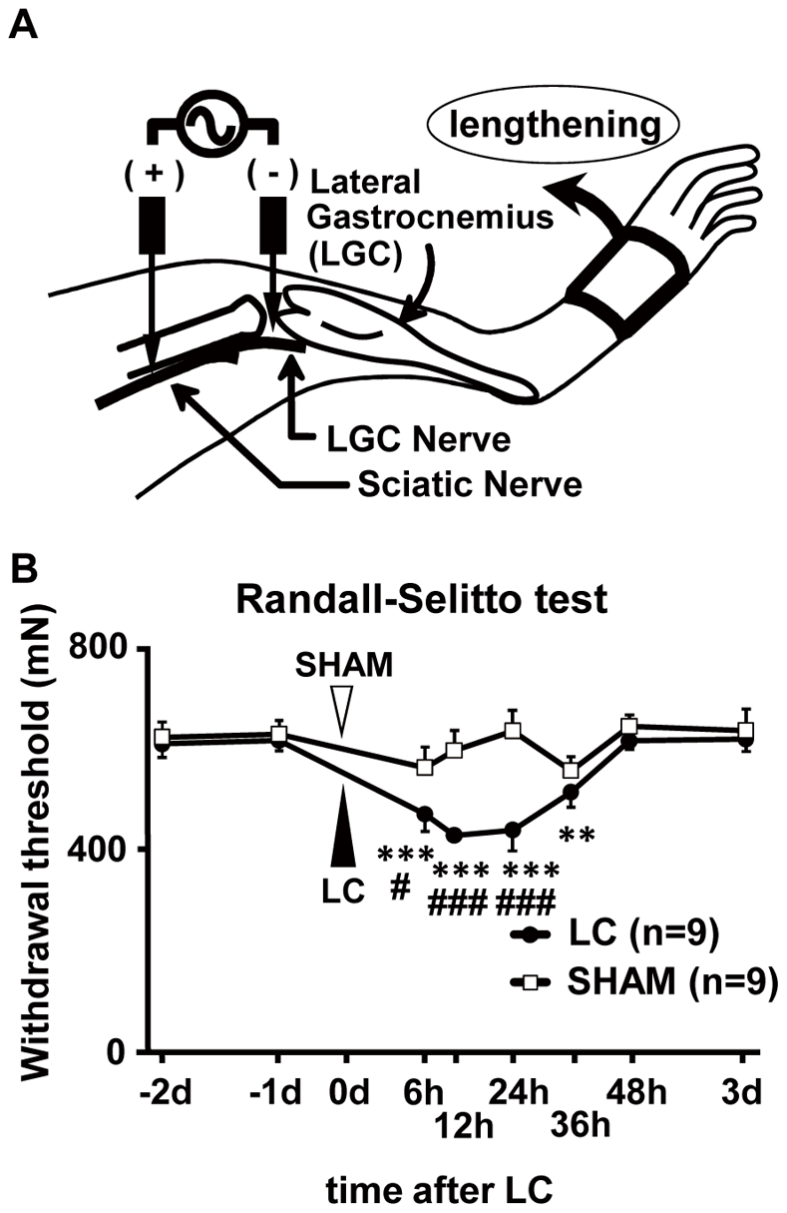
Muscular mechanical hyperalgesia induced by lengthening contraction in mice. (**A**) Schema of lengthening contraction (LC) application to the lower hindleg flexors, mainly the lateral gastrocnemius (LGC) muscle. LC was induced by electrical stimulation through a pair of needle electrodes inserted near the tibial and sciatic nerves. The ankle joint was dorsi-flexed in synchrony with muscle contraction, and then returned to the starting position over a 3 s resting period. This cycle was repeated 300 times. (**B**) Change in withdrawal thresholds by RST in WT mice that received LC or sham (stretch only) exercise (n = 9 for each group, mean ± S.E.M.). Vertical axis: withdrawal threshold in mN, horizontal axis: time after exercise. There was a significant difference between the groups, and the threshold decreased 6 to 36 h after exercise in LC group, but not in sham group. ** *p*<0.01, *** *p*<0.001 compared with −1 day in LC group; # *p*<0.05, ### *p*<0.001 compared with sham group on each time point, two-way repeated measures ANOVA with Bonferroni t-test.

Next, we examined whether DOMS develops in mice similar to rats. The mechanical withdrawal threshold measured by RST was almost stable before exercise, at 629.2±26.2 mN for the sham exercised animals (sham group) and 616.4±19.7 mN for LC animals (LC group) 1 day before exercise. It remained at a similar level in the sham group during the entire period of observation. LC was applied as schematically shown in Fig. 2A. The threshold in the LC group started to decrease 6 h after exercise, and reached a minimum 12–24 h after exercise (Fig. 2B). Complete recovery was observed 48 h after exercise. The change in the threshold in the LC group was statistically significant 6, 12, 24, and 36 h after exercise compared with that 1 day before exercise (−1d in Fig. 2B, *p*<0.01–0.001, Bonferroni t-test). The threshold of the LC group was significantly lower than the sham group 6, 12, and 24 h after exercise (*p*<0.05–0.001), thus, mice became mechanically hyperalgesic after LC. Despite the decreases in the threshold, no spontaneous pain-related behaviors such as licking, biting, or lifting of the exercised hindleg were observed, and the mice walked normally in the cage up to the end of experiment. Therefore, we concluded that mechanical hyperalgesia after LC can be used as a DOMS model also in mice.

### LC did not Induce DOMS in TRPV1−/− and TRPV4−/− Mice

**Figure 3:**
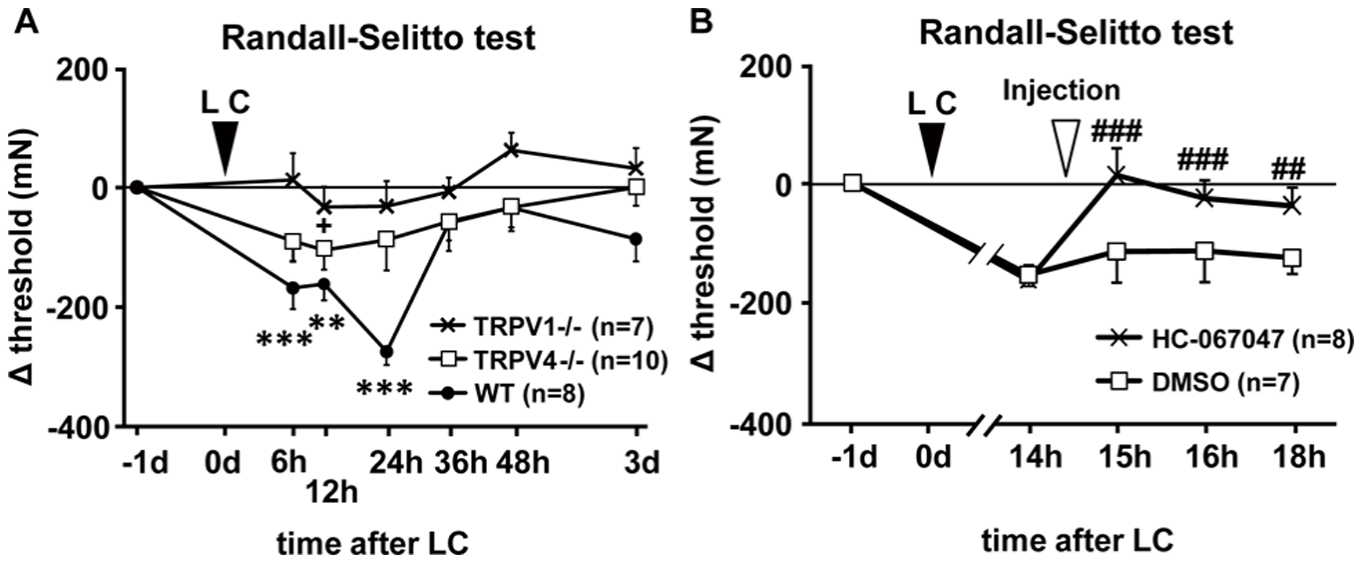
Muscular mechanical hyperalgesia did not develop after LC in TRPV1−/− and TRPV4−/− mice. (**A**) Change in the withdrawal thresholds after LC measured by RST in TRPV1−/− (crosses) and TRPV4−/− (open squares) mice. Vertical axis: difference in the threshold from −1 d in mN, horizontal axis: time after LC. (**B**) Changes in the mechanical hyperalgesia by intramuscular injection of HC-067047, a TRPV4 selective antagonist (100 mg/kg; crosses) or DMSO (open squares) in WT mice. Mean ± S.E.M. (n = 6–10 for each group). ** *p*<0.01, *** *p*<0.001 compared with −1 d in WT,+*p*<0.05 compared with −1 d in TRPV4−/−, ## *p*<0.01, ### *p*<0.001 compared with 14 h in HC-067047 group; two-way repeated measures ANOVA with Bonferroni t-test.

Using the TRPV1−/− and TRPV4−/− mice, we examined possible involvement of TRP channels in DOMS. There were no differences in the baseline RST thresholds before exercise among groups (one-way ANOVA): on day −1, the threshold in TRPV1−/−, TRPV4−/−, and wild-type (WT) was 630.6±26.3, 626.1±20.8, and 630.4±22.0 mN, respectively. The change from day −1 is plotted along time after LC in Fig. 3A. A two-way repeated measures ANOVA with main factors of time and genotype showed a significant decrease in time (*p*<0.001), a significant difference in genotypes (*p<*0.01), and a significant interaction between these two factors (F_2, 6_ = 2.246, *p*<0.05). Post hoc analysis showed a significant difference between TRPV1−/− and WT mice (*p*<0.001, Bonferroni t-test) but no significant difference between TRPV4−/− and WT (*p* = 0.227, Bonferroni t-test) or TRPV1−/− (*p* = 0.152). When compared with the −1 day value, there was a significant decrease at 6–24 h in WT, but no difference in either TRPV1−/− or TRPV4−/− mice (except 12 h after LC). These results suggest that both TRPV1 and TRPV4 play a pivotal role in mechanical hyperalgesia after LC.

Despite involvement of TRPV1 in DOMS was pharmacologically investigated [33], involvement of TRPV4 has not been pharmacologically investigated. Therefore, we tested whether HC-067047 [40], a TRPV4 antagonist, reduces the threshold decrease after LC. The thresholds 1 day before LC (−1 d) in HC-067047 and vehicle (dimethyl sulfoxide, DMSO) injection groups were 658.4±17.1 mN and 635.2±24.7 mN, respectively, and not different each other. When clear decrease in the withdrawal threshold was observed 14 h after LC, the antagonist was intramuscularly injected and its effect was recorded. A two-way repeated measures ANOVA with main factors of time and drug showed a significant decrease in time (*p*<0.001), a significant difference in drug (*p<*0.05), and a significant interaction between these two factors (F_1, 7_ = 2.246, *p*<0.05). Post hoc analysis showed a significant recovery of the withdrawal threshold in the HC-067047 group at 15, 16, and 18 h after LC when compared with that before injection (0.5, 1.5, and 3.5 h after injection of the drug: *p*<0.001, *p*<0.01, and *p*<0.01, Bonferroni t-test, Fig. 3B). In contrast, no change was observed in the vehicle group (Fig. 3B). These results suggest that TRPV4 is involved in the mechanical hyperalgesia after LC.

### LC up-regulated NGF mRNA in Both TRPV1−/− and TRPV4−/− Mice

**Figure 4:**
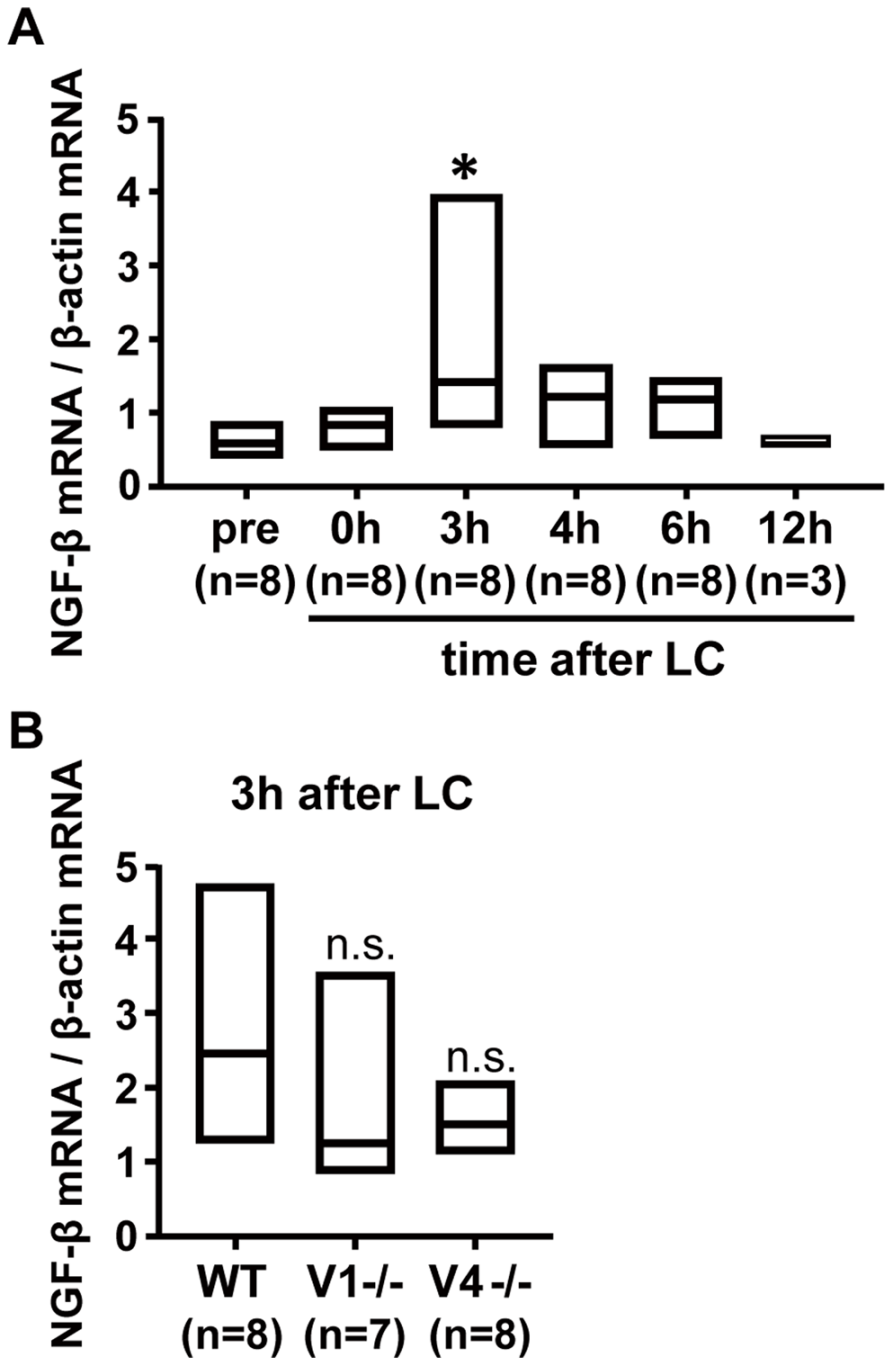
NGF-β mRNA was up-regulated in all three genotypes. (**A**) Time course of NGF-β mRNA expression in LC-exercised LGC muscle in WT mice. (**B**) Up-regulation of NGF-β mRNA 3 h after LC in the muscle of three genotypes. Median and interquartile range (IQR). All values were normalized with β-actin mRNA. n = 3–8 for each group (shown in the parentheses under each column). * *p*≤0.05 compared with pre, and n.s. not different from WT (Kruskal-Wallis one-way analysis of variance on ranks test followed by the Dunn's test).

Previously we reported that NGF is up-regulated in the exercised muscle after LC and plays a pivotal role in DOMS [4]. To determine whether NGF up-regulation occurs also in TRPV1−/− and TRPV4−/− mice, we checked NGF-β mRNA level (value normalized with β-actin mRNA) in the muscle using real-time PCR. Before measurements in knockout mice, we checked the time course of the change in NGF-β mRNA level in WT mice. NGF-β mRNA of the exercised muscle showed no change shortly after LC (0 h in Fig. 4A). Three hours after LC a clear increase was observed (Fig. 4A). Therefore, we measured the NGF-β mRNA level in knockout mice 3 h after exercise. NGF-β mRNA levels in TRPV1−/− and TRPV4−/− mice were not significantly different from the WT mice 3 h after exercise (Fig. 4B, with Kruskal-Wallis one-way analysis of variance on ranks test followed by the Dunn's test). It must be noted that the normalized level of NGF-β mRNA was not different among genotypes before exercise: 0.8 (IQR: 0.4–1.1), 0.9 (IQR: 0.7–1.2), and 0.7 (IQR: 0.6–1.2) in WT, TRPV1−/−, and TRPV4−/−, respectively. Thus, we concluded that the up-regulation process of NGF expression in the exercised muscle is impaired neither in TRPV1−/− nor TRPV4−/− mice.

### NGF did not Induce Mechanical Hyperalgesia in TRPV1−/− Mice

**Figure 5:**
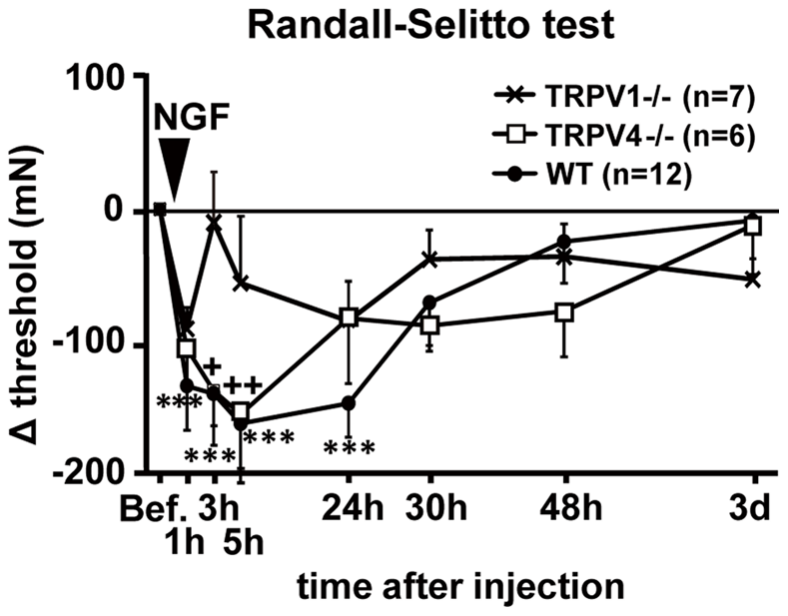
Muscular mechanical hyperalgesia was not developed after NGF injection in TRPV1−/− mice. Change in the withdrawal threshold by intramuscular injection of NGF-2.5 S was measured by RST. Vertical axis: difference in the withdrawal threshold from that before NGF-injection, horizontal axis: time after injection. WT mice (closed circles), TRPV1−/− mice (crosses) and TRPV4−/− mice (open squares). *** *p*<0.001 compared with −1 d (Bef. in the figure) in WT;+*p*<0.05,++*p*<0.01 compared with −1 d in TRPV4−/−; two-way repeated measures ANOVA with Bonferroni t-test.

Next, we examined whether mechanical hyperalgesia develops as a result of intramuscular injection of NGF in TRPV1−/− and TRPV4−/− mice to investigate their contribution to downstream of NGF up-regulation in DOMS. The baseline RST thresholds before NGF injection in TRPV1−/−, TRPV4−/−, and WT mice were 667.7±16.4, 551.4±29.4, and 628.8±25.1 mN, respectively, and there was no significant difference among genotypes (one-way ANOVA). NGF injection induced mechanical hyperalgesia in WT and TRPV4−/− mice but not in TRPV1−/− mice (Fig. 5). Statistical analysis showed there were significant differences among genotypes (*p*<0.05) and times (*p*<0.001), but no significant interaction between genotype and time (F_2,7_ = 1.656, *p* = 0.070, two-way repeated measures ANOVA). Significant mechanical hyperalgesia was detected in WT mice 1, 3, 5, and 24 h after injection (*p*<0.001, Bonferroni t-test in Fig. 5). In TRPV1−/− mice the threshold did not significantly decrease after NGF injection (Fig. 5), while the threshold in TRPV4−/− mice decreased after NGF injection with a time course very similar to that of WT mice (significant decrease at 3 and 5 h after injection, Bonferroni t-test, *p*<0.01–0.05, Fig. 5).

### LC did not Up-regulate GDNF mRNA in TRPV4−/− Mice

**Figure 6:**
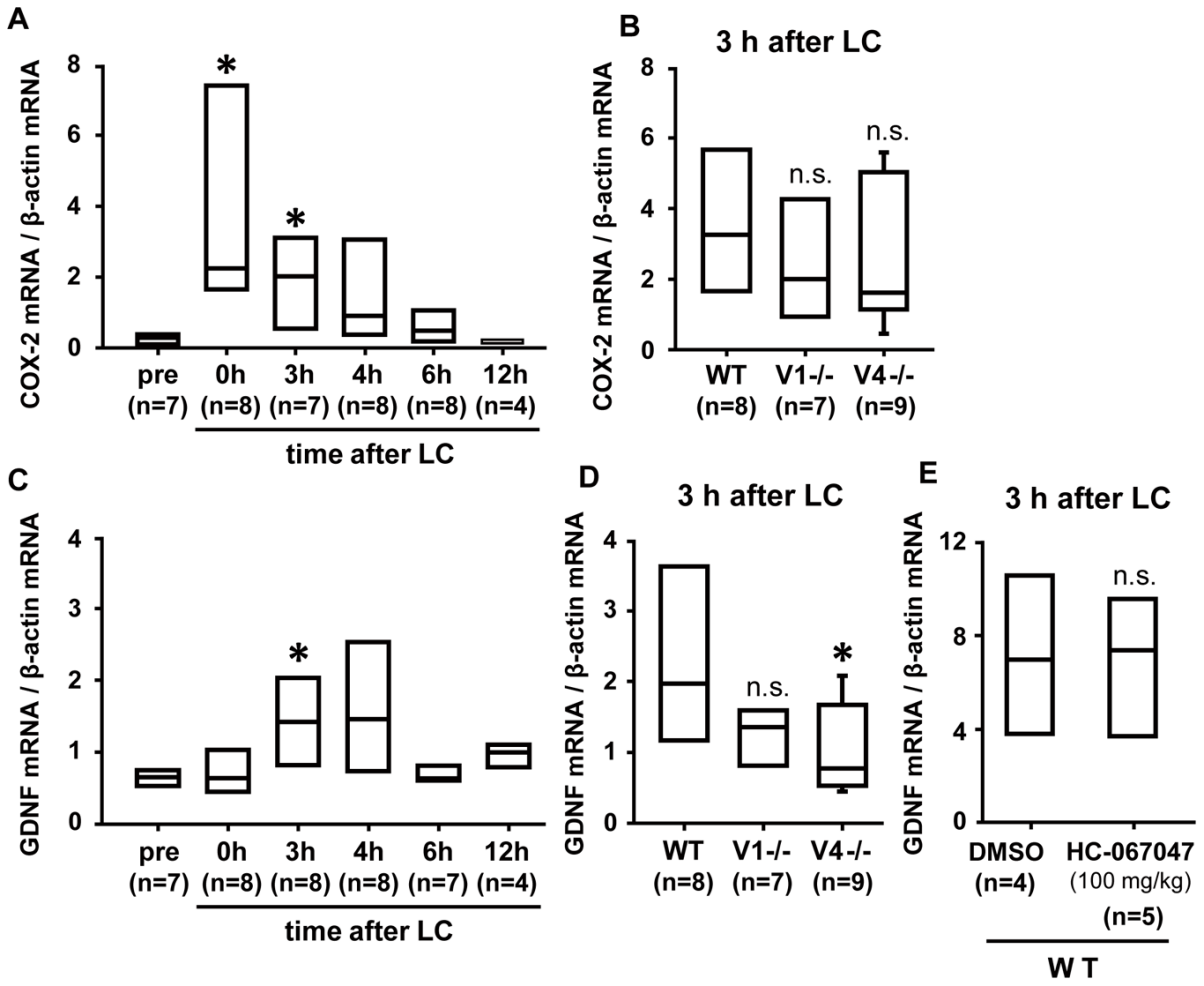
Change in expression levels of COX-2 and GDNF mRNA after LC in TRPV1−/− and TRPV4−/− mice. (**A**) COX-2 mRNA level in excised LGC muscle of WT mice after LC. (**B**) Up-regulation of COX-2 mRNA 3 h after LC in the muscle of three genotypes. (**C**) GDNF mRNA level in excised LGC muscle of WT mice. (**D**) Up-regulation of GDNF mRNA 3 h after LC in the muscle of three genotypes. (**E**) Effect of HC-067047 on the level of GDNF mRNA 3 h after LC. All values were normalized with β-actin mRNA. Median and interquartile range are shown. Number of animals used is shown in the parentheses under each column. * *p*≤0.05 compared with pre (in C) and with WT (in D); n.s., not different from WT in B and D, or from DMSO group in E, by Kruskal-Wallis one-way analysis of variance on ranks test followed by the Dunn's test except in E. Mann-Whiteny U test was used in E.

We have previously shown that in addition to NGF, GDNF plays a pivotal role in generation of DOMS, and that COX-2 up-regulation is essential (COX-2– GDNF pathway) for GDNF up-regulation [5]. To know whether GDNF up-regulation occurs also in TRPV1−/− and TRPV4−/− mice, we checked in WT mice the time course of COX-2 mRNA up-regulation similarly to NGF. COX-2 mRNA expression started to increase 0 h after LC and the increase lasted up to 3 h after LC in WT (Fig. 6A). Previously, we showed that the increase in COX-2 mRNA and protein 0 h after LC was not specific to LC among different forms of contraction, but that the later increase was specific [5]; therefore, we investigated whether COX-2 mRNA increased 3 h after LC in TRPV1−/− and TRPV4−/− mice. COX-2 mRNA was found to increase after LC in these knockout mice, too (Fig. 6B). The normalized level of COX-2 mRNA before LC was not different among genotypes: 0.4 (IQR: 0.2–0.6), 0.3 (IQR: 0.3–0.4) and 0.3 (IQR: 0.3–0.4) in WT, TRPV1−/−, and TRPV4−/−, respectively.

Next, we checked GDNF mRNA level in TRPV1−/− and TRPV4−/− mice. First we examined the time course of GDNF mRNA up-regulation in WT mice. Different from COX-2 mRNA but similar to NGF mRNA, no up-regulation was observed 0 h after LC (Fig. 6C). Three hours after the exercise, the level of GDNF mRNA was significantly higher than that before LC (pre) (Fig. 6C, *p*<0.05). Therefore, we investigated the GDNF level in knockout mice at the time point of 3 h after LC. The GDNF mRNA level of the exercised LGC in TRPV1−/− was not significantly different from WT mice (Fig. 6D). In contrast, the level in TRPV4−/− mice was significantly lower than WT mice (Fig. 6D). The normalized level of GDNF mRNA before LC was not different among genotypes: 0.7 (IQR: 0.6–0.9), 0.8 (IQR: 0.8–0.9) and 0.6 (IQR: 0.5–0.7) in WT, TRPV1−/−, and TRPV4−/−, respectively.

To further investigate the involvement of TRPV4 in the upstream of GDNF mRNA expression, we injected HC-067047 0.5 h before and shortly after LC. However, the expression level was not different between vehicle- and antagonist-treated groups at doses, 10 or 100 mg/kg (only the result with 100 mg/kg is shown in Fig. 6E).

### GDNF Induced Mechanical Hyperalgesia in Neither TRPV1−/− Nor TRPV4−/− Mice

**Figure 7:**
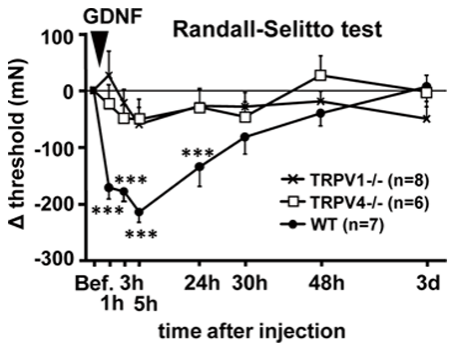
GDNF failed to induce muscle mechanical hyperalgesia both in TRPV1−/− and TRPV4−/− mice. Change in the withdrawal threshold by intramuscular injection of GDNF was evaluated by RST. Vertical axis: difference in withdrawal threshold from that before GDNF-injection, horizontal axis: time after the injection. The threshold in WT mice (closed circles) decreased 1 to 24 h after the injection, while that in TRPV1−/− (crosses) or TRPV4−/− mice (open squares) did not decrease. Mean ± S.E.M. Number of animals used is in the parentheses. *** *p*<0.001 compared with −1 d (Bef in the graph) in WT; two-way repeated measures ANOVA with Bonferroni t-test.

Finally, we examined possible involvement of these TRPV channels in GDNF-induced mechanical hyperalgesia. We injected GDNF into the LGC muscle and measured the withdrawal threshold by RST. The withdrawal threshold before injection in TRPV1−/−, TRPV4−/−, and WT was 639.0±26.1, 607.3±13.1, and 635.9±20.6 mN, respectively, not significantly different from each other (one-way ANOVA). WT mice showed clear mechanical hyperalgesia after GDNF injection, but neither TRPV1−/− nor TRPV4−/− mice did. Statistical analysis showed significant effects in time (*p*<0.001) and genotype (*p*<0.05), and significant interaction between genotype and time (F_2, 7_ = 4.681, *p*<0.001, two-way repeated measures ANOVA, one factor repetition). Both TRPV1−/− and TRPV4−/− were different from WT (*p*<0.05). In WT, GDNF induced mechanical hyperalgesia 1, 3, 5, and 24 h after injection (*p*<0.001, Bonferroni t-test, Fig. 7). Thus, the effect of GDNF in WT mice was shorter than in rats, similar to LC or NGF. In contrast to WT, the withdrawal threshold in TRPV1−/− and TRPV4−/− mice did not decrease after GDNF injection (Fig. 7). These results demonstrate that GDNF, which is up-regulated in the skeletal muscle similarly to NGF, induces mechanical hyperalgesia through both TRPV1 and TRPV4 activation.

## Discussion

In the current study, we validated a method for measuring muscular mechanical hyperalgesia in mice, and found DOMS and up-regulation of NGF and GDNF after LC appear earlier (DOMS, 6 h after LC; NGF/GDNF up-regulation, 3 h after LC) and disappear earlier (DOMS, 24–36 h after LC; NGF/GDNF up-regulation, 6 or 12 h after LC) than in rats. In addition we demonstrated an involvement of TRPV1 and TRPV4 in DOMS, but in different way.

### Time Course of DOMS in Mice

The peak of hyperalgesia after LC was observed at 12–24 h, which is half shorter than in rats [3]. Again, the recovery was faster than in rats [3], and the mechanical threshold returned to the baseline level in 36–48 h after LC. Similarly, up-regulation of NGF and GDNF was observed 3 h after LC, faster than in rats (12 h), but there was still a delay [4], [5]. In contrast, there is not much difference between rats and humans in the time course of development of DOMS [2], [3]. What determines this difference or similarity in DOMS time course is not known.

### Interaction of TRPV1 with NGF in Mechanical Hyperalgesia

We found that DOMS did not develop in TRPV1−/− and TRPV4−/− mice, but NGF mRNA level increased 3 h after LC in both genotypes as well as in WT mice. In this experiment, we did not measure the level of NGF protein, but we consider that NGF protein was also up-regulated because we observed its up-regulation in a previous experiment [4]. These results show the NGF up-regulating pathway is not impaired in either TRPV1−/− or TRPV4−/− mice. These channels are expressed in nociceptors [17], [34], thus they are possibly sensitized after exercise. This hypothesis is supported with the previous results that capsazepine reduced mechanical hyperalgesia after LC [33] and the present result that a specific antagonist for TRPV4 reduced mechanical hyperalgesia after LC.

The present observation showed that the mechanical threshold of TRPV1−/− mice was not different from that of WT under normal conditions, but the NGF injection that induced mechanical hyperalgesia in WT mice failed to induce mechanical hyperalgesia in TRPV1−/− mice. Murase et al. [4] previously reported that NGF sensitizes muscle nociceptors to mechanical stimulation, and TRPV1 is reported to be expressed in small DRG neurons [17]. Together with the present result, it thus seems highly likely that NGF sensitizes TRPV1 expressed in the muscular thin-fiber afferents. There are several reports regarding interaction between NGF and TRPV1. NGF is reported to sensitize the response to TRPV1 agonist capsaicin through activation of protein kinase A [41] or protein kinase C [42]. NGF is also known to act on TrkA receptor to increase membrane expression of TRPV1 by activating PI3 kinase and then Src kinase, resulting in rapid sensitization [43]. It is also reported that NGF increases the number of TRPV1 expressing neurons in mouse trigeminal ganglion neurons in inflammatory heat hyperalgesia [44], but these latter two processes may take longer than the first one. All these reports studied the effects of NGF either on capsaicin response or heat response, but not on mechanical response.

Involvement of TRPV1 in mechanical sensitivity has been reported for afferents of the jejunum [45] and colon [46]. In addition, involvement of TRPV1 in mechanical hyperalgesia has been known in the skin [47], viscera [48] and muscle [32], as reported here. Since direct activation of TRPV1 channel by mechanical event is not yet known, other mechanisms must be considered. One possibility is the production of endogenous agonists by mechanical stimulation such as N-arachidonoyl-dopamine [49], [50] and N-oleoyl-dopamine [51], [52], which might activate/sensitize TRPV1. Alternatively, TRPV1 may be somehow connected with a mechanotransducing channel, and thereby contribute to mechanotransduction. The latter probability was suggested in a paper reporting sensitization of mechanosensitive currents by protons from the authors’ group [53].

In TRPV4−/− mice the mechanical threshold decreased after NGF injection, similar to WT mice (Fig. 5), and NGF mRNA up-regulation after LC was not different from WT mice. These results suggest that TRPV4 is involved in neither NGF-induced mechanical hyperalgesia nor up-regulation of NGF.

### Interaction Between COX-2– GDNF Pathway and TRPV1/TRPV4

The contribution of TRPV1 and TRPV4 to the COX-2– GDNF pathway seems to be a little more complex. First, in both TRPV1−/− and TRPV4−/− mice, the COX-2 mRNA level increased 3 h after LC similarly to the WT mice. Therefore, neither TRPV1 nor TRPV4 acts upstream or in the process of COX-2 mRNA up-regulation. GDNF mRNA at 3 h after LC was significantly lower in TRPV4−/− mice than WT mice, but that in TRPV1−/− mice was not different from WT. However, the specific TRPV4 antagonist HC-067047 failed to reduce up-regulation of GDNF mRNA. This is not due to insufficient dosage of the antagonist, because mechanical hyperalgesia after LC was reduced with the same dosage. One possibility explaining this discrepancy might be that HC-067047 could block the site of TRPV4 involved in nociceptor sensitization by GDNF but could not block the site involved in up-regulation of GDNF mRNA in the muscle as pointed out for TRPV1 [54]. There are reports suggesting functional expression of TRPV4 in skeletal muscle [28]–[30], but no direct evidence of channel activation. Therefore TRPV4 might be involved in the process of GDNF up-regulation after COX-2 activation in DOMS. The prostaglandin receptors responsible for DOMS are expressed in the muscle [55], and so it is speculated that the ionic changes produced by TRPV4 activation in the muscle during LC might have modulated signaling after prostaglandin receptor activation. Alternatively, there might be a yet unknown pathway involving TRPV4 that is operated parallel to COX-2 activation and leads to up-regulation of GDNF. This point is open for future study.

In TRPV1−/− mice the mechanical threshold after GDNF injection did not decrease, suggesting that TRPV1 is involved in GDNF-mediated mechanical hyperalgesia. This result is different from an observation from our lab that GDNF-induced mechanical hyperalgesia was not blocked by capsazepine in rats [56], but in agreement with a report by Malin et al. [57] that showed GDNF sensitized muscular DRG neuron responses and increased nociceptive behavior to capsaicin in mice. Although capsazepine is a competitive antagonist of capsaicin, and the capsaicin binding site, heat sensor area, and proton interaction area in the TRPV1 channel are different (for review see [54]), it has been reported to block proton response (in humans but less effectively in rats) [58], heat hyperalgesia [59] and mechanical hyperalgesia [60]. The differences in these pharmacological experiments with capsazepine and experiments in TRPV1−/− mice might be the result of species difference, or alternatively from the fact that TRPV1−/− mice lack TRPV1 not only in the peripheral nervous system but also in the central nervous system. It is now known that TRPV1 is expressed in GABAergic interneurons in the spinal cord and plays important roles in controlling pain sensitivity, because its activation resulted in a reduction of inhibitory signaling to spinothalamic tract projection neurons [61]. Thus it is plausible that global loss of TRPV1 might make animals resistant to hyperalgesia.

Similarly, GDNF failed to induce mechanical hyperalgesia in TRPV4−/− mice. The cascade by which GDNF activates TRPV4 has not been explored. Following activation of Ret, the GDNF receptor that works with coreceptors GFRα1 or 2, PLCγ/IP3 is activated [62]. Sensitization of the TRPV4 channel by IP3 has been reported [63]. This cascade might be involved in the sensitization of TRPV4 by GDNF.

TRPV4 is known to being activated by shear or hypoosmotic stress in extracellular fluid in endothelial [64], urothelial [65], and other cells. Stretching the cell membrane by hypoosmotic stress liberates arachidonic acid inside the cell and activates TRPV4 via eicosatrienoic acid [66], [67]. These reports together suggest that mechanical change of the sarcolemma during LC might possibly activate TRPV4, leading to up-regulation of GDNF. This point is also open for future study.

### Conclusion

We developed a mouse model of DOMS and showed that it is useful in investigating the mechanism of DOMS or mechanical hyperalgesia in general. Using knockout mice we found that TRPV1 contributes to DOMS downstream of NGF and GDNF, while TRPV4 is located downstream of up-regulating GDNF and possibly also its upstream.

## Materials and Methods

### 1) Experimental Animals

Adult male C57Bl/6J mice were used in this study, comprising 124 WT (Charles River Laboratories Inc., Japan), 34 TRPV1−/−, and 36 TRPV4−/− mice. This study consisted of four experiments. First was to validate the method for measuring the mechanical withdrawal threshold of the deep tissues in mice (n = 19). Second was to establish a method of exercise to induce DOMS in mice (n = 18). Third was to measure changes in hyperalgesic behavior and muscular genes (NGF, GDNF and COX-2) in WT, TRPV1−/− and TRPV4−/− mice subjected to LC (n = 111). Last was to evaluate contribution of TRPV1 and TRPV4 ion channels to NGF- or GDNF-induced mechanical hyperalgesia (n = 46).

The mice were kept three to four per cage under a 12 h light/dark cycle (light between 07.00 h and 19.00 h) in an air-conditioned room (22–24°C). They had food and water ad libitum throughout the experiment. All experiments were conducted according to the Regulations for Animal Experiments in Nagoya and Chubu Universities, the National Institute for Physiological Sciences, and the Fundamental Guidelines for Proper Conduct of Animal Experiments and Related Activities in Academic Research Institutions in Japan.

### 2) Withdrawal Threshold Measurement

#### 2.1. Randall-Selitto test (RST)

A Randall-Selitto apparatus (Ugo Basile, Comerio, Italy) was used to measure the withdrawal threshold of the deep tissues, mainly the lateral head of the gastrocnemius (LGC) muscle. The animals were restrained with a towel around the trunk to calm them, and treated gently during experiments. A cone-shaped pusher with a rounded tip (tip diameter: 2.6 mm) was applied to the belly of the LGC muscle through shaved skin. The speed of the application force was set at 78 mN/s (slower than for rats) and the cut-off point was set at 1049 mN to avoid damaging the tissue. The intensity of the pressure that caused an escape reaction was defined as the withdrawal threshold, decrease of which was regarded as a sign of mechanical hyperalgesia. Training sessions were carried out for at least one week. Measurements were performed four times at about 90 s intervals and the mean value of the last three trials was taken as the threshold. Each series of the experiments was done at almost the same period of time during the day to avoid circadian fluctuations. Experimenters were blind to which exercise or reagents the mice received.

#### 2.2. von frey hair test (VFT)

The mechanical withdrawal threshold of the skin over the LGC muscle was measured with self-made von Frey hairs (VFHs, tip diameter: 0.25 mm, bending forces 27.12–1027.46 mN in quasi-logarithmic order) because the mechanical strain induced by thin VFHs barely reaches the deeper muscle layer [68]. The mice were restrained at the trunk with a towel, similar to the RST. Each filament was applied to the skin two times at intervals of a few seconds. The threshold was determined by the method of limits (briefly, changing the forces of VFHs up and down), and if an animal showed at least one withdrawal response, this was taken as a positive response.

#### 2.3. Local anesthesia and induction of inflammatory hyperalgesia

To validate the method of measuring deep mechanical hyperalgesia through skin, we evaluated our method under surface anesthesia. In human studies 30 min application of EMLA® cream (containing prilocaine 25 mg and lidocaine 25 mg in 1 g, AstraZeneca Inc., London, UK) reportedly blocks nociceptive afferents up to 1–2 mm from the surface [69]. We therefore applied EMLA cream to the shaved skin over the LGC muscle for 30 min and then removed it with ethanol. Ointment cream without anesthetics was applied (vehicle control group) similar to EMLA cream.

Inflammation was induced by injecting 40 µl of 3% lambda carrageenan (Type IV, Sigma, St. Louis, MO, USA) dissolved in sterile saline into the LGC muscle through a 30 G thin injection needle [70]. Mechanical withdrawal threshold was measured by both VFT and RST 12 h before carrageen injection and on the next day before and after the EMLA treatment.

### 3) Exercise Protocol

The method of LC for mice in this study was almost the same as previously reported for rats [3] and only some points were modified. Briefly, the animals were anesthetized with halothane or isoflurane (1.0–2.0%). LC was induced in the lower hind leg flexors (mainly LGC muscle) by electrical stimulation of the tibial nerve through a pair of needle electrodes inserted near the nerve (Fig. 2A). Electrical stimulation with the following parameters was applied for 1 s: current strength of three times the twitch threshold (<100 µA), and a frequency of 50 Hz with pulse duration of 1 ms. The ankle joint was dorsi-flexed in synchrony with muscle contraction with use of a linearized servomotor (Oriental Motor Co. Ltd., Japan) to stretch the LGC muscle, and then returned to the starting position over a 3 s resting period. This cycle was repeated 300 times. LC applied only to the right leg, and the left leg was used as a control. A passive stretch without electrically-stimulated contraction was used for sham exercise in the experiment for establishing the LC method. After the animals recovered from anesthesia, they behaved, ate and drank normally.

Withdrawal threshold was measured by the Randall-Selitto apparatus at 6, 12, 24, 36 and 48 h and 3 d after LC.

### 4) Injection of NGF, GDNF and HC-067047

Murine NGF-2.5 S (0.8 µM, 5 µl, i.m. dissolved in 0.01 M PBS) was injected into the belly of the LGC muscle of the right hindlimb under halothane or isoflurane anesthesia (1.4–2.0%, Abbott, Chicago, Illinois, USA). NGF was obtained from two companies, Sigma and Wako (Osaka, Japan). We confirmed that these substances had almost the same effect, namely, inducing the same level of decrease in withdrawal threshold (data not shown). Recombinant murine GDNF (0.03 µM, 5 µl, i.m. dissolved in 0.01 M PBS; Peprotech, Rocky Hill, USA) was also used. Withdrawal threshold was measured by RST before injection, and 1, 3, 5, 24, 30, 48 h, and 3 d after injection.

For pharmacologically examining the involvement of TRPV4, its antagonist HC-067047 (10 or 100 mg/kg, 10 µl, i.m. dissolved in dimethyl sulfoxide, DMSO; Tocris Bioscience, Bristol, UK) was used. To examine whether TRPV4 is involved in the development of DOMS, it was injected into the same muscle two times, 0.5 h before and shortly after LC, then the muscle was dissected 3 h after LC, and GDNF mRNA was measured. For examining whether TRPV4 is involved in mechanical hyperalgesia, this antagonist (100 mg/kg) was injected 14.5 h after LC when the mechanical hyperalgesia was clear. The RST threshold was measured one day before and 14, 15, 16, and 18 h after LC. For the control vehicle (DMSO) was injected instead of the antagonist.

### 5) Measurement of mRNA

LGC muscle was removed under anesthesia immediately after LC (0 h) and 3, 4, 6, and 12 h after LC. Tissue was weighed, frozen in liquid nitrogen and stored at −80°C. From 30 mg of each sample, total RNA was extracted with RNeasy® Fibrous Tissue Mini Kit (Qiagen, Hilden, Germany). One µg of total RNA was reversetranscribed with M-MLV Reverse Transcriptase (Promega, Madison, USA) at 42°C for 50 min. The aliquot of cDNA derived from 30 ng of total RNA was used as a template for a single PCR reaction. Quantitative real time RT-PCR was done with Power SYBR® Green PCR Kit (Life Technologies, Carlsbad, USA) and run on Rotor-Gene™ Q (Qiagen). For each target gene, samples were run with a diluted series of mice muscle cDNA as standards once in triplicate. The primers were: forward 5′-CCTCCAATCCTGTTGAGAGTG-3′ and reverse 5′-TGTGAGTCGTGGTGCAGTATG-3′ for NGF-β, forward 5′-CCTGCTGCCCGACACCTTCA-3′ and reverse 5′-AGCAACCCGGCCAGCAATCT-3′ for COX-2, forward 5′-CCAGAGAATTCCAGAGGGAAAGGT-3′ and reverse 5′-TCAGTTCCTCCTTGGTTTCGTAGC-3′ for GDNF, and forward 5′-CCATCCTGGCCTCACTGT-3′ and reverse 5′-CCGGACTCATCGTACTCCTG-3′ for β-actin. Samples were initially denatured at 95°C for 10 min, amplified by 40 cycles at 95°C for 15 s and at 60°C for 60 s, and followed by the melting analysis from 60 to 95°C. Threshold cycle (Ct) and relative expression level to the standards were determined by Rotor-Gene™ Q series software 1.7 (Qiagen). β-actin was measured as an internal standard and the two-standard curve method was used for normalization. NGF, COX-2, and GDNF mRNAs were normalized with β-actin mRNA. Outliers were omitted by the Smirnov-Grubbs' outlier test.

### 6) Statistical Analysis

The data from the RST and mRNA measurement were presented as mean ± S.E.M. Change in withdrawal threshold during the observation period was examined two-way ANOVA with repeated measures followed by the Bonferroni t-test, compared with the threshold on day −1 or in WT. The change of mRNA in exercised muscle was presented as median and interquartile range (IQR), and examined with Kruskal-Wallis one-way analysis of variance on ranks test followed by the Dunn's test. The Mann-Whitney test was used to compare GDNF mRNA expression in the WT and TRPV4−/− mice.

The data from VFT were presented as median and IQR. The results with carrageenan and EMLA cream were analyzed by the Mann-Whitney test.

*p*<0.05 was considered to indicate a significant change.
